# Stigma and its influencing factors in female patients with high-risk HPV infection: a cross-sectional study

**DOI:** 10.3389/fpsyt.2025.1613755

**Published:** 2025-06-27

**Authors:** Lili Gong, Xiaonan Li, Dan Hou

**Affiliations:** ^1^ Department of Psychiatry and Psychology, General Hospital of Northern Theater Command, Shenyang, Liaoning, China; ^2^ Department of Health Management, General Hospital of Northern Theater Command, Shenyang, Liaoning, China

**Keywords:** human papillomavirus infection, stigma, psychological resilience, rumination, influence factor

## Abstract

**Background:**

Stigma associated with high-risk human papillomavirus (HR-HPV) infection presents a significant barrier to both the uptake of cancer screening and the completion of follow-up care after a positive screening result. Identifying the factors that contribute to this stigma is essential for healthcare professionals to design targeted interventions aimed at reducing stigma and improving patient engagement across the continuum of care.

**Objectives:**

This exploratory study aimed of this study is to explore the current situation of stigma in HR-HPV patients and its influencing factors.

**Method:**

A cross-sectional study was performed from May to October 2023. Two hundred female patients with HR-HPV infection were recruited from the Department of Gynecology at the General Hospital of Northern. Demographic parameters, disease-related variables, psychosocial factors, psychological resilience, and rumination data were obtained by self-reported questionnaires.

**Results:**

Of the 200 patients, 37 (18.5%) indicated a low degree of stigma, 152 (76.0%) indicated a moderate level, and 11 (5.5%) indicated a high level. Stigma scores exhibited a positive correlation with rumination scores and a negative correlation with psychological resilience scores. Monthly personal income, recurring infections, psychological resilience, and rumination were recognized as independent variables affecting stigma. This exploratory study revealed that psychological resilience and rumination collectively explained 23.4% of the variance in stigma.

**Conclusion:**

Personal monthly income, recurring infections, psychological resilience, and rumination were influential factors contributing to stigma in HR-HPV patients. Subsequent research should prioritize the development and integration of interventions aimed at eliminating stigma.

## 1 Introduction

The prevalence of human papillomavirus (HPV) infection among Chinese women varies by region, with estimates ranging from 9.6% to 23.6% (1). High-risk HPV (HR-HPV) infections can endure and are strongly associated with the development of several malignancies, including cervical, anal, vaginal, vulvar, penile, and oropharyngeal cancers. Receiving a diagnosis of HR-HPV may have a significant psychological effect on women. Abnormal cytology results are often linked to feelings of sadness, anxiety, trouble sleeping, changes in sexual behavior, and delusional thoughts. Furthermore, women with HR-HPV frequently experience anxiety around the disclosure of their diagnosis, which may threaten their emotional relationship with their partners (2). These results underscore the imperative of attending to the mental health needs of women diagnosed with HR-HPV.

Stigma, which is when people have negative thoughts and feelings about certain health problems, can make it much harder for people to agree to cancer screening and follow-up treatment after a positive diagnosis. This issue is particularly salient in the context of HR-HPV, as the stigma surrounding the condition often stems from its sexual transmission and preventability. Studies conducted in low- and middle-income countries demonstrate that HPV is frequently linked to conceptions of promiscuity, prostitution, and infidelity, resulting in anxieties of blame, social ostracism, and relationship strain (3). Consequently, stigma around HPV has been correlated with reduced vaccine acceptability and delayed participation in cancer screening, notwithstanding the availability of these service.

Psychological resilience, a fundamental concept in positive psychology, is characterized as the capacity to maintain one’s dedication to life objectives and existential purposes notwithstanding challenges (4). People who are more resilient are more flexible, which helps them deal with stressors better and improves their mental and physical health (5). Following an HR-HPV diagnosis, patients with high psychological resilience are more likely to display positive emotions and an optimistic outlook, allowing them to adjust more rapidly and mitigate the emotional distress associated with the infection (6). Emerging evidence supports a link between psychological resilience and reduced stigma (5, 7–9); however, the nature of this relationship in patients with HR-HPV remains unclear. Rumination, by contrast, refers to repetitive, prolonged, and intrusive negative thoughts focused on the self, emotions, personal concerns, and distressing experiences (10). It tends to intensify and prolong negative emotional states such as sadness, anger, anxiety, and depression. Previous studies have shown that rumination plays a significant role in the development of self-stigma among individuals engaged in hazardous drinking (11). To date, however, the potential effect of rumination on stigma in patients with HR-HPV has not been investigated.

Identifying the factors that influence stigma can help healthcare professionals develop targeted strategies to reduce stigma among patients with HR-HPV infection. However, limited research has examined these influencing factors within the context of Chinese culture. Therefore, this study aimed to investigate the current status of stigma and examine the effects of demographic characteristics, disease-related variables, psychosocial factors, psychological resilience, and rumination on stigma in patients with HR-HPV.

## 2 Material and methodology

### 2.1 Study design and participants

A cross-sectional survey was conducted from May to October 2023 to recruit female outpatients diagnosed with HR-HPV at the Department of Gynecology, General Hospital of Northern Theater Command, Shenyang, Liaoning Province, China. Participants were selected through convenience sampling. HPV DNA testing was performed using a genotyping kit capable of detecting 23 HPV types through PCR-reverse dot blot analysis. This assay identifies 17 high-risk types (HPV 16, 18, 31, 33, 35, 39, 45, 51, 52, 53, 56, 58, 59, 66, 68, 73, and 82) and six low-risk types (HPV 6, 11, 42, 43, 81, and 83). A positive result for high-risk HPV indicates the presence of at least one of the 17 high-risk genotypes in the DNA sample.

Inclusion criteria for participants were (1): aged >18 years old, with a history of sexual activity (2), patients with no abnormalities in cervical liquid-based cytology, colposcopy, and pathological examination results. The exclusion criteria included (1) pregnancy (2), suffering from immune and metabolic diseases (3); suffering from psychiatric disorders or serious somatic disease (4), having suffered a major traumatic event in the past 6 months.

### 2.2 Ethics consideration

This study does not involve human harm or distress. The study was approved by the ethics committee of the General Hospital of Northern Theater Command (approval no. ethical review -Y-2023–111). The informed consent form signed by all patients before enrollment.

### 2.3 Measurement

The level of stigma was assessed using the Chinese version of the Social Impact Scale (SIS) (12, 13). The SIS consists of 24 items across four dimensions: social rejection (9 items), financial insecurity (3 items), internalized shame (5 items), and social isolation (7 items). Each item is rated on a 4-point Likert scale ranging from 1 (“strongly disagree”) to 4 (“strongly agree”), yielding total scores from 24 to 96, with higher scores indicating greater stigma. Stigma levels were divided into low (1.00–1.99), moderate (2.00–2.99), and high (3.00–4.00) based on the average item score. The Cronbach’s alpha coefficient for the SIS in this study was 0.928, which means that the internal consistency was quite high.

The Chinese version of the Connor-Davidson Resilience Scale (CD-RISC) was employed to assess psychological resilience. The CD-RISC, developed in 2003, evaluates traits such as adaptation and stress resilience in both clinical and general populations. The Chinese version has undergone rigors cross-cultural adaptation and validation, confirming its suitability for Chinese populations (14–16). This validated instrument has demonstrated substantial reliability in cross-cultural research and is widely employed in mental health studies. The CD-RISC consists of 25 items evaluated on a 5-point Likert scale, from 0 (“not at all true”) to 4 (“almost always true”). Higher scores indicate greater psychological resilience. The study determined that the Cronbach’s alpha coefficient for the CD-RISC was 0.968, signifying outstanding internal consistency.

The Chinese Event-Related Rumination Inventory (C-ERRI) is a validated psychological tool designed to assess rumination tendencies following traumatic or stressful events in Chinese individuals. The C-ERRI, created through careful cross-cultural adaptation, has two parts: intrusive rumination and deliberate rumination. Each subscale consists of 10 items rated on a 4-point Likert scale, from 1 (“never”) to 4 (“often”), to measure how often ruminative thoughts occurred in the past month. The inventory demonstrates strong psychometric properties, including sufficient internal consistency and test-retest reliability, with its bifactorial structure confirmed using confirmatory factor analysis. The overall score can be anywhere from 20 to 80, and higher values suggest more rumination (17). The Cronbach’s alpha coefficient for the C-ERRI in this study was 0.920.

### 2.4 Study size

According to Riley’s sample size calculation method (18), the required sample size should be 5 to 10 times the number of variables, resulting in a range of 110 to 219 participants. To ensure adequate statistical power, we set a confidence level of 95% and an acceptable margin of error of 5%. Based on these parameters, the minimum required sample size was determined to be 200. The present study included 200 patients with HR-HPV, thereby meeting the calculated sample size requirements.

### 2.5 Statistical analysis

Data analysis was conducted using SPSS version 22.0. The Kolmogorov–Smirnov test was applied to assess the normality of the data distribution. Measurement data were expressed as mean ± standard deviation (M±*SD*). Group comparisons were performed using analysis of variance (*t/F* test). Pearson correlation analysis was conducted to examine relationships among SIS, CD-RISC, and C-ERRI scores. Hierarchical regression analysis was used to identify factors influencing stigma, with age and marital status included as covariates (*α*_enter_ = 0.05, *α*_remove_ = 0.10). Multicollinearity among predictor variables was assessed using the Variance Inflation Factor (VIF), where a VIF value greater than 10 indicated severe multicollinearity, warranting corrective actions such as removing or combining correlated variables. A significance level of *P* < 0.05 was set for all analyses.

## 3 Results

### 3.1 Study sample characteristics

During the study period, approximately 287 female patients with HR-HPV infection were eligible, of whom 200 agreed to participate, resulting in a participation rate of about 67%. None of the patients were infected with human immunodeficiency virus (HIV). **Table 1** presents the detailed demographic characteristics of the 200 female patients. The mean age was 39.60 ± 9.87 years. Among them, 61.50% had a single HPV infection, while 38.50% had multiple infections. The majority were first-time infections, accounting for 83.00%, with recurrent infections comprising 17.00%.

**Table 1:** Participants’ characteristics (n = 200).

Variables	n	%
Age (Year)		
18-29	28	14.00
30-44	116	58.00
45-59	33	16.50
≥60	23	11.50
Nationality		
Han	169	84.50
Other	31	15.50
Marital status		
Married	162	81.00
Unmarried	38	19.00
Education background		
Primary or below	32	16.00
Junior/Senior high school	46	23.00
College degree or above	122	61.00
Religious belief		
Yes	17	8.50
No	183	91.50
Employment		
Employed	151	75.50
Unemployed	49	24.50
Hospital payment		
Medical insurance	156	78.00
Self payment	44	22.00
Personal monthly income		
≤3000 RMB	33	16.50
3001~4999 RMB	61	30.50
≥5000 RMB	106	53.00
Live alone		
Yes	41	20.50
No	159	79.50
Habitation		
Urban	166	83.00
Countryside	34	17.00
Procreation		
Yes	151	75.50
No	49	24.50
Duration of HPV infection (T)		
1 month ≤ T ≤ 3 months	56	28.00
3 months <T ≤ 1 year	67	33.50
T > 1 year	77	38.50
Type of HPV infection		
Single infection	123	61.50
Mixed infection	77	38.50
Recurrent infection		
Yes	34	17.00
No	166	83.00

### 3.2 The current status and univariate analysis of stigma in patients with HR-HPV

**Figure 1:**
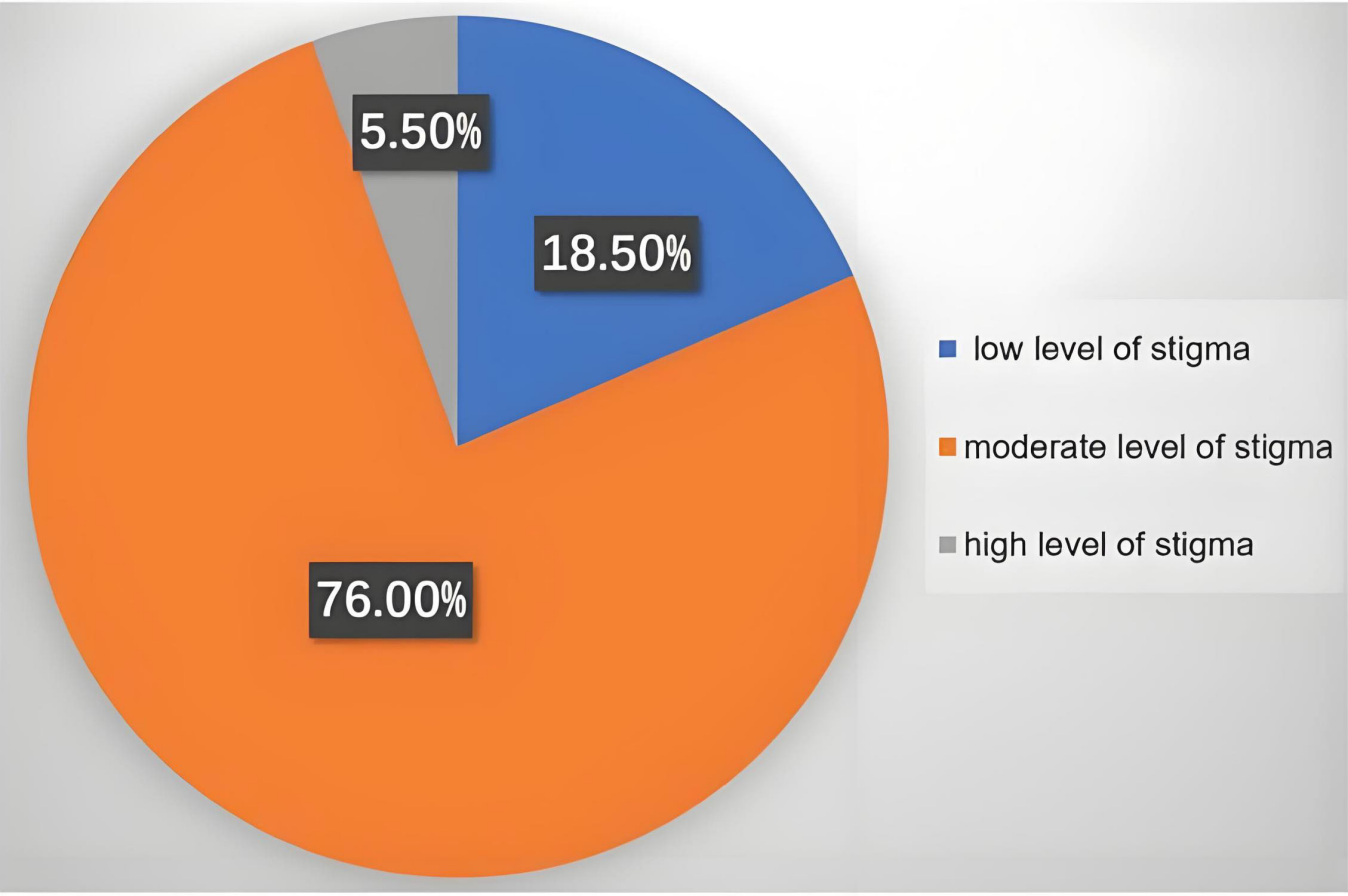
The levels of stigma in 200 patients with HR-HPV.

The dimensions of SIS were ranked from lowest to highest based on average item scores as follows: financial insecurity, social rejection, social isolation, and internalized shame. Among the 200 patients, 37 (18.5%) exhibited a low level of stigma, 152 (76.0%) a moderate level, and 11 (5.5%) a high level of stigma (**Table 2**; **Figure 1**). **Table 3** presents the distribution of stigma scores among patients with HR-HPV. Significant differences were found in stigma scores across different levels of personal monthly income (*P* < 0.05). Additionally, patients with recurrent infections had higher stigma scores compared to those without recurrent infections (*P* < 0.05).

**Table 2:** The SIS scores of 200 patients with HR-HPV.

Variables	Total average score (M±*SD*)	Item average score (M±*SD*)	low level of stigma (n, %)	moderate level of stigma (n, %)	high level of stigma (n, %)
Social rejection	19.76 ± 4.01	2.19 ± 0.53	53 (26.50)	135 (67.50)	12 (6.00)
Financial insecurity	5.85 ± 2.01	1.95 ± 0.67	134 (67.00)	56 (28.00)	10 (5.00)
Internalized shame	12.33 ± 3.70	2.47 ± 0.84	54 (27.00)	109 (54.50)	37 (18.50)
Social isolation	16.29 ± 3.20	2.31 ± 0.50	30 (15.00)	151 (75.50)	19 (9.50)
Total score	53.26 ± 10.07	2.23 ± 0.39	37 (18.50)	152 (76.00)	11 (5.50)

**Table 3:** Distribution of stigma scores with different demographic characteristics (n = 200).

Variables	The SIS scores (M±*SD*)	*F/t*	*P*
Age (Year)		2.037	0.110
18-29	51.96 ± 12.02		
30-44	54.74 ± 10.96		
45-59	53.70 ± 11.73		
≥60	52.00 ± 12.17		
Nationality		1.021	0.308
Han	53.22 ± 11.27		
Other	55.68 ± 12.50		
Marital status		0.757	0.449
Married	53.80 ± 11.80		
Unmarried	52.39 ± 9.96		
Education background		2.086	0.127
Primary or below	56.14 ± 12.20		
Junior/Senior high school	55.09 ± 13.70		
College degree or above	52.32 ± 10.19		
Religious belief		1.665	0.097
Yes	58.06 ± 9.69		
No	53.01 ± 11.55		
Employment		0.986	0.326
Employed	53.03 ± 11.00		
Unemployed	55.04 ± 12.82		
Hospital payment		0.506	0.613
Medical insurance	53.03 ± 11.50		
Self payment	54.02 ± 11.49		
Personal monthly income		5.378	0.005
≤3000 RMB	59.45 ± 12.37		
3001~4999 RMB	52.44 ± 11.55		
≥5000 RMB	52.44 ± 10.65		
Live alone		0.159	0.873
Yes	53.00 ± 12.62		
No	53.35 ± 11.19		
Habitation		0.523	0.601
Urban	53.07 ± 11.45		
Countryside	54.21 ± 11.61		
Procreation		0.747	0.456
Yes	52.98 ± 11.65		
No	54.35 ± 11.00		
Duration of HPV infection (T)		0.114	0.892
1 month ≤ T ≤ 3 months	52.73 ± 12.41		
3 months < T ≤ 1 year	54.03 ± 11.58		
T > 1 year	53.13 ± 10.77		
Type of HPV infection		1.213	0.226
Single infection	52.15 ± 11.57		
Mixed infection	54.14 ± 11.12		
Recurrent infection		2.120	0.035
Yes	56.53 ± 12.02		
No	51.79 ± 11.12		

### 3.3 The correlations between stigma, psychological resilience and rumination

The mean C-ERRI score of the 200 patients with HR-HPV was 20.80 ± 10.01. The mean CD-RISC score for these patients was 58.99 ± 16.77, which was significantly lower than that of the Chinese general population (65.40 ± 13.90, *P* < 0.05) (4). The total SIS score was positively correlated with the C-ERRI score (r = 0.634, *P* < 0.001) and negatively correlated with the CD-RISC score (r = -0.586, *P* < 0.001).

### 3.4 Multivariate stratified linear regression analysis of stigma in patients with HR-HPV

The VIF values for all independent variables in our hierarchical regression analysis were below 10, indicating no multicollinearity concerns. **Table 4** presents the results of multivariate hierarchical linear regression analysis exploring factors influencing stigma, with the total SIS score as the dependent variable. In Model 1, personal monthly income and recurrent infection status were included as independent variables. Model 2 further incorporated CD-RISC and C-ERRI scores, which showed statistically significant correlations. Psychological resilience and rumination together explained 23.4% of the variance in stigma (*P* < 0.05) (**Table 5**). Rumination had a positive effect on stigma (*β* = 0.355, *P* < 0.001), whereas psychological resilience had a negative effect (*β* = -0.097, *P* = 0.037).

**Table 4:** Multivariate stratified linear regression analysis of stigma in patients with HR-HPV.

Variables	B	SE	*β*	*t*	*P*
Model 1					
Constant	46.974	1.748	–	26.865	<0.001
Personal monthly income	-2.38	1.068	-0.156	-2.228	0.027
Nonrecurrent infection	5.146	2.131	0.169	2.414	0.017
Model 2					
Constant	43.898	3.362	–	13.059	<0.001
Personal monthly income	-1.018	0.968	-0.067	-1.052	0.294
Nonrecurrent	2.639	1.876	0.087	1.407	0.161
Psychological resilience	0.355	0.054	0.434	6.599	<0.001
Rumination	-0.097	0.046	-0.142	-2.105	0.037

**Table 5:** Summary results of the hierarchical regression analysis model.

Model	R	R^2^	Adjusted R^2^	*F*	*P*
1	0.249	0.062	0.052	6.475	0.002
2	0.548	0.300	0.286	33.260	0.001

## 4 Discussion

Few previous studies have examined the factors influencing stigma in patients with HR-HPV. In this study, we investigated the current status of stigma among HR-HPV patients and identified its influencing factors. Our findings indicate that patients experience varying levels of stigma, with moderate stigma being the most common. Specifically, personal monthly income, recurrent infection, psychological resilience, and rumination were found to be significant factors affecting stigma.

Stigma was originally defined as a social process that categorizes individuals with perceived physical or moral flaws as possessing a “tainted identity,” distinguishing them from those considered “normal” (19). This process can be divided into self-stigmatization and social stigmatization, which interact to create barriers for individuals seeking help (20). Among patients with HPV, stigma arises primarily because the infection is sexually transmitted and preventable (21). Previous studies suggest that HPV-related stigma contributes to low HPV vaccine uptake, even when resources are available (22). Moreover, stigma in patients with HR-HPV acts as a barrier to cancer screening and hinders completion of the continuum of care after a positive screening result (23). In our study, stigma was assessed using SIS, which has demonstrated good reliability across different populations (24). In the present study, 37 patients (18.5%) exhibited low levels of stigma, 152 (76.0%) moderate levels, and 11 (5.5%) high levels. These findings underscore the importance of identifying factors influencing stigma in patients with HR-HPV.

In the present study, multivariate stratified linear regression analysis revealed that personal monthly income, recurrent infection, psychological resilience, and rumination were significant factors influencing stigma. Numerous previous studies have identified economic income as an important factor affecting stigma (25–27). Individuals with higher income levels generally have greater social resources, enabling them to better overcome adversity and experience lower levels of depression and anxiety (4). Conversely, families with low monthly income face more challenges in managing the process of seeking HPV treatment, which may lead to higher affiliate stigma and difficulties in coping (28). Notably, lower income levels have also been negatively associated with awareness of the HPV vaccine (29). Our study found that patients with recurrent HPV infection had higher stigma scores compared to those without recurrence, possibly due to the economic burden and negative emotions associated with repeated infection (30). Given the recurrent nature of HR-HPV infection, accurate and timely screening is essential for effective control. Furthermore, targeted interventions addressing stigma in recurrent patients warrant increased attention.

Psychological resilience refers to the ability to maintain one’s orientation toward existential purpose despite facing adversities and stressful events (4). There is a close relationship between psychological resilience and stigma. Individuals with high psychological resilience tend to accept their disease with a positive attitude, actively confront negative emotions, and experience lower levels of stigma (31). In contrast, those with low resilience often respond to difficulties through avoidance, which can exacerbate the burden of disease and increase stigma (31, 32). Our study confirmed that psychological resilience negatively impacts stigma. Supporting this, previous research has shown that resilience influences the quality of life and depressive symptoms in diabetes patients through stigma (32). Rumination is a form of perseverative thinking characterized by repetitive and unproductive focus on distressing events (33). It intensifies and prolongs negative emotional states such as anxiety and depression (34). Among individuals living with sexually transmitted diseases, rumination can have debilitating effects, especially when combined with stress (35). Rumination and self-stigma may be closely linked, as both involve negative patterns of thinking. However, research on the relationship between rumination and stigma in patients with HR-HPV remains scarce. Our study found that rumination positively influences stigma.

Psychological resilience and rumination independently accounted for 23.4% of the variance in stigma. Our exploratory findings suggest that enhancing psychological resilience and reducing rumination could be effective strategies to mitigate stigma among HR-HPV patients. However, further research is needed to confirm these associations. Interventions such as resilience training—particularly those incorporating mindfulness and cognitive-behavioral techniques—may strengthen resilience (36). For rumination, cognitive behavioral therapy, mindfulness-based interventions, and attentional shifting have demonstrated efficacy in alleviating symptoms (10, 37, 38).

While our study identified personal monthly income, recurrent infection, psychological resilience, and rumination as key factors influencing stigma among HR-HPV patients, it is crucial to consider the potential impact of cultural factors. Traditional Chinese values such as modesty, collectivism, and the emphasis on family honor may shape patients’ perceptions and coping strategies regarding their HR-HPV diagnosis. These cultural norms can contribute to reluctance in disclosing their status, fear of social rejection, and internalized shame, thereby intensifying stigma. Additionally, the stigma surrounding sexually transmitted infections like HR-HPV may be aggravated by cultural beliefs that associate such infections with moral judgments (3). In some societies, HR-HPV is associated with promiscuity or moral deficiencies, hence exacerbating stigmatization (39). Future research should explore the distinct cultural influences on stigma among HR-HPV patients in China and analyze how these cultural factors interact with the socio-demographic and psychological variables revealed in this study.

## 5 Clinical practice implications

(1) Enhanced Screening and Follow-Up Programs : ① Personalized Screening Strategies : Because repeated illnesses and a person’s monthly income have a big impact on stigma, healthcare practitioners should tailor their screening and follow-up plans to each patient’s financial situation and history of infections. This may include more frequent screenings and specialized mental health help for people with limited incomes or chronic conditions. ② Resilience Training Programs : Add techniques that help people with HR-HPV be more resilient to their regular care. Mindfulness-based stress reduction, cognitive-behavioral therapy, or group sessions designed to enhance coping methods and foster positive psychological adaptability may constitute effective approaches (40). Psychosocial Support Services : ① Offer counselling and psychotherapy that is easy to receive and not too expensive, and that specifically deals with the mental health problems that come with an HR-HPV diagnosis, such as rumination and stigma. These services must be culturally appropriate and available to patients of all income levels. ② Peer Support Groups : Encourage the establishment of peer support groups for patients to share experiences, exchange coping skills, and offer mutual emotional support. This community-oriented strategy helps mitigate feelings of isolation and reduce stigma (2). Patient Education and Awareness : ① Formulate and execute educational programs that encompass both the medical aspects of HR-HPV and its psychological and social implications. These initiatives should emphasize the significance of early detection, accessible treatment alternatives, and efficient stigma management. ② Media Campaigns : Utilize media venues to enhance public knowledge on HR-HPV, highlighting its treatability and the essential importance of screening. Such campaigns can aid in dispelling misconceptions and diminishing societal stigma associated with the virus.

## 6 Limitation of the study

This study has several limitations. First, the use of a convenience sampling method may have introduced selection bias, limiting the generalizability of our findings to the broader population of HR-HPV patients. Although this approach allowed for efficient recruitment, it may have excluded patients with more severe symptoms or those from different socioeconomic backgrounds. Future studies should consider employing random sampling or other more representative techniques to minimize bias and improve external validity. Second, due to the exploratory nature of this study and the lack of precise effect size estimates in this context, an *a priori* power analysis was not conducted; future research should incorporate power calculations based on preliminary data or existing literature. Third, the cross-sectional design prevents any causal inferences regarding the associations observed, and longitudinal studies are needed to elucidate causal relationships. Additionally, unmeasured confounding factors such as family history of cancer, social support networks, and previous healthcare experiences may have influenced patients’ stigma perceptions and psychological resilience, and should be accounted for in future research. Fourth, this study did not evaluate negative emotions, which may mediate the impact of stigma, warranting further investigation. Fifth, data were collected via self-report, a method susceptible to subjectivity and potential bias. Sixth, the sample was geographically limited to Liaoning Province, China, which may restrict the applicability of findings; future studies should include more diverse, multi-regional samples. Finally, only female patients were included, excluding males despite evidence that stigma may vary by gender (41); future research should explore these gender differences.

## 7 Conclusion

This study indicates that HR-HPV patients recruited from a tertiary hospital in northeastern China experience varying levels of stigma, with moderate stigma being the most common. Personal monthly income, recurrent infection, psychological resilience, and rumination were identified as significant factors influencing stigma in this population. While these findings offer important insights, future research should incorporate more diverse samples across multiple regions and healthcare settings to enhance the generalizability of the results. Moreover, longitudinal studies are warranted to elucidate the causal relationships between these factors and stigma.

## Data Availability

The original contributions presented in the study are included in the article/supplementary material. Further inquiries can be directed to the corresponding author.
